# Bile Acid Stress Disrupts Homeostasis and Placental Functional Programs in Human First-Trimester Trophoblast Organoids

**DOI:** 10.3390/ijms272110023

**Published:** 2026-11-09

**Authors:** Shutian Zhang, Li Li, Sili Zheng, Qing Xu, Zhida Qian, Jingjing Su, Weibin Wu

**Affiliations:** 1 International Peace Maternal and Child Health Hospital, Shanghai Jiao Tong University School of Medicine, Shanghai 200030, China; stya001@sjtu.edu.cn (S.Z.);; 2 Shanghai Key Laboratory of Embryo Original Diseases, Shanghai 200030, China; 3 Shanghai Ninth People’s Hospital, Shanghai Jiao Tong University School of Medicine, Shanghai 200011, China

**Keywords:** intrahepatic cholestasis of pregnancy, trophoblast organoids, bile acids, placental trophoblast, trophoblast homeostasis, transcriptomics

## Abstract

Intrahepatic cholestasis of pregnancy (ICP) is characterized by elevated maternal bile acids (BAs), yet their effects on human trophoblast organization and transcriptional responses remain incompletely understood. Here, we used human first-trimester trophoblast organoids (TOs) to characterize cellular and transcriptional responses to a defined six-component BA mixture (50–150 μM). BA exposure disrupted organoid architecture and intercellular organization, with attenuated proliferation-associated features and more prominent SDC1 staining. These changes were accompanied by increased DNA fragmentation and cleaved caspase-3 staining and by reduced ATP-dependent viability, supporting an apoptotic component of BA-associated trophoblast injury. Bulk RNA sequencing after exposure to 150 μM BA for 96 h identified 413 differentially expressed genes, of which 364 were downregulated and 49 were upregulated. Downregulated transcripts were primarily associated with placental endocrine and secretory processes, epithelial organization, cell adhesion, and transport, whereas a smaller subset of lipid-handling genes was upregulated. RT-qPCR confirmed concordant upregulation of PLIN2 and ABCG1 in two independent donor-derived TO lines. Collectively, these findings show that BA mixture exposure is associated with structural injury and altered transcriptional profiles in human TOs, thereby providing insight into trophoblast responses to BA stress.

## 1. Introduction

Intrahepatic cholestasis of pregnancy (ICP) is the most common pregnancy-specific liver disorder, affecting approximately 0.5–5% of pregnancies worldwide, with a particularly high prevalence in East Asian and Latin American populations [1]. ICP is commonly diagnosed on the basis of maternal pruritus and elevated serum total bile acid (TBA) concentrations and typically presents during the second or third trimester [2,3]. Although maternal symptoms generally resolve after delivery, ICP is associated with increased risks of adverse perinatal outcomes, including preterm birth, meconium-stained amniotic fluid, and fetal growth abnormalities [4,5]. Notably, in singleton pregnancies, the risk of stillbirth increases substantially when peak maternal TBA concentrations reach or exceed 100 μmol/L [6]. However, the mechanisms linking elevated maternal TBA levels to placental dysfunction and adverse fetal outcomes remain incompletely understood.

The placenta is the principal interface between the maternal and fetal circulations and performs essential endocrine, transport, metabolic, and barrier functions required for fetal growth and pregnancy maintenance [7]. Trophoblasts largely mediate these functions. Villous cytotrophoblasts (CTBs) proliferate, differentiate, and fuse to form the multinucleated syncytiotrophoblast (STB), which constitutes the outer surface of the placental villi, regulates maternal–fetal exchange, and secretes hormones and pregnancy-associated proteins [7,8]. Disruption of CTB proliferation, differentiation, or STB functional maturation may therefore compromise placental homeostasis [8,9]. As an important interface for maternal–fetal bile acid transport, the placenta is exposed to elevated bile acid concentrations in ICP, which may disrupt trophoblast function and cause cellular injury [10,11]. Experimental evidence indicates that excessive BAs can activate inflammatory signaling, impair autophagic flux, and induce endoplasmic reticulum and mitochondrial stress in trophoblasts [11,12,13,14]. However, most previous studies have relied on immortalized trophoblast cell lines, including HTR-8/SVneo and BeWo cells, cultured in conventional two-dimensional systems. Such models may not fully reproduce the three-dimensional architecture, polarized organization, cell–cell interactions, and progenitor dynamics of the human placental trophoblast compartment.

The establishment of human trophoblast organoids (TOs) by Turco et al. represented an important advance in experimental placental biology [15]. Derived from first-trimester villous cytotrophoblasts, TOs form self-organizing three-dimensional epithelial structures that retain a TP63α-positive progenitor-like population and can differentiate toward STB and extravillous trophoblast lineages under defined culture conditions [15,16]. Subsequent methodological refinements have enabled their reproducible establishment and application across laboratories [17]. Compared with conventional trophoblast cell lines, TOs more faithfully preserve trophoblast lineage organization and CTB-to-STB differentiation within a three-dimensional epithelial architecture. They have therefore been increasingly used to investigate trophoblast progenitor maintenance, lineage differentiation, maternal–fetal interactions, and placental barrier function [17,18]. Trophoblast stem cell- and organoid-based models have also been applied to disease-associated placental phenotypes, including preeclampsia and recurrent pregnancy loss [19,20]. However, the application of this model to ICP-associated placental injury remains limited. In particular, the effects of mixed-BA exposure on organoid architecture, CTB-associated features, cellular injury, and transcriptional profiles have not been adequately characterized in a human three-dimensional trophoblast model.

In this study, TOs established from first-trimester donors were exposed to a six-component BA cocktail. We first assessed BA-associated structural alterations and ATP-dependent viability and then performed transcriptomic and molecular analyses to characterize the cellular response. This study aimed to determine how BA stress affects organoid architecture, CTB-associated features, cellular injury, viability, and placental function-associated transcriptional programs.

## 2. Results

### 2.1. Establishment and Characterization of Human Placental Trophoblast Organoids

**Figure 1:**
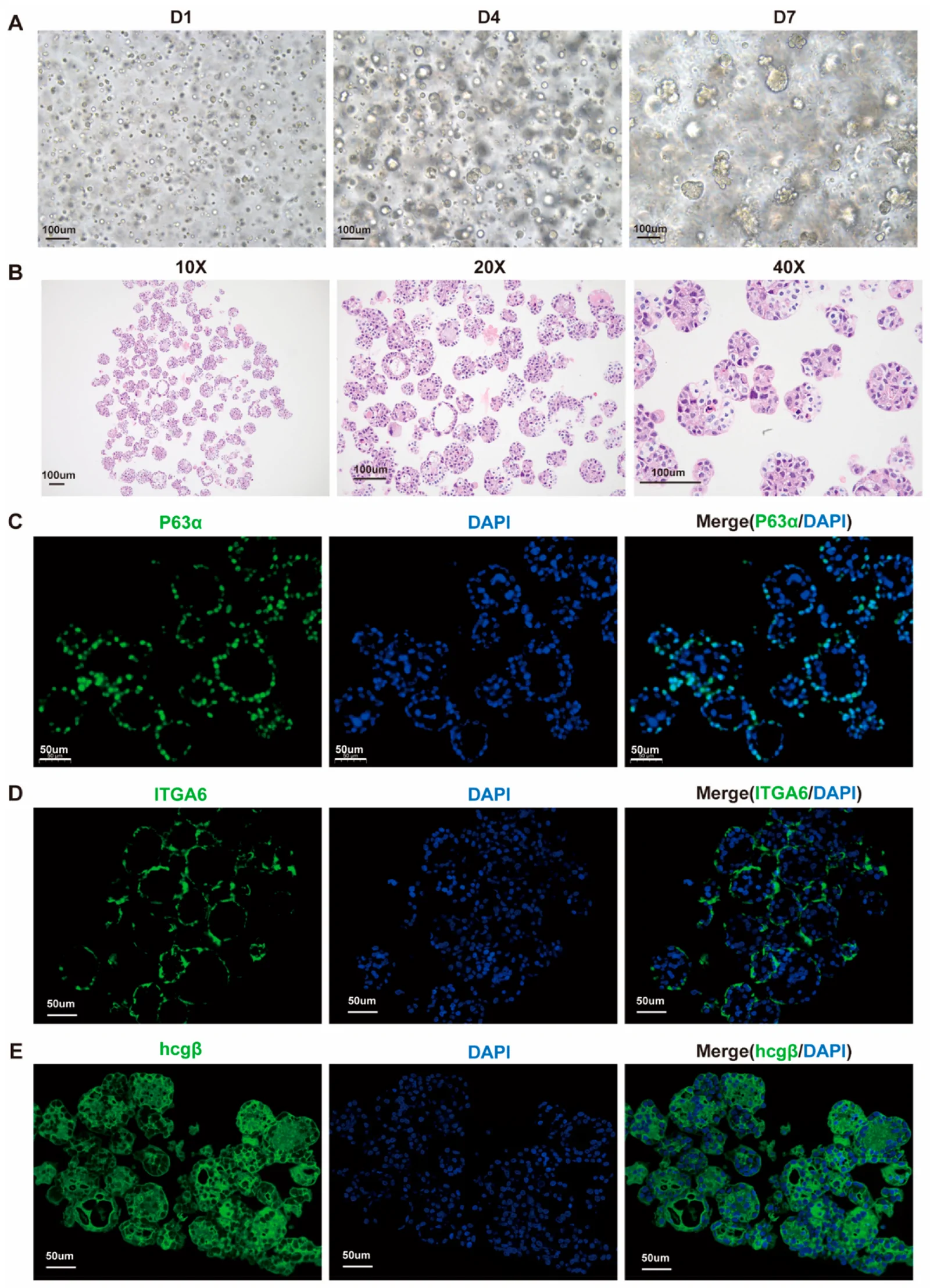
Establishment and characterization of human first-trimester trophoblast organoids (TOs). (**A**) Representative bright-field images of TO line 1# acquired using a 10× objective, showing progressive organoid formation and growth on days 1, 4, and 7 after passage. Scale bars, 100 μm. (**B**) Representative hematoxylin and eosin (H&E)-stained sections showing organoid histology, acquired using 10×, 20×, and 40× objectives. Scale bars, 100 μm. (**C**–**E**) Representative immunofluorescence images of the villous cytotrophoblast (CTB)-associated markers p63α (**C**) and ITGA6 (**D**) and the syncytiotrophoblast (STB)-associated marker hCGβ (**E**), acquired using a 63× objective. Markers are shown in green and DAPI-counterstained nuclei in blue. Scale bars, 50 μm.

TOs were established from first-trimester placental villi obtained from two independent healthy donors. We assessed organoid formation, histological architecture, and lineage-associated marker expression. p63α is highly expressed in proliferative villous CTBs and contributes to maintenance of the CTB progenitor state [15,21]. Bright-field microscopy showed progressive organoid formation over 7 days of culture in a representative line (1#; Figure 1A). Small cellular aggregates on day 1 developed into compact spheroidal and cystic structures with lumen-like regions by days 4–7. H&E staining showed organized multicellular spheroids with cohesive epithelial-like layers and variable central lumen-like spaces (Figure 1B). Immunofluorescence analysis revealed characteristic trophoblast lineage features: p63α and ITGA6 marked CTB-associated populations, whereas hCGβ and SDC1 supported STB-associated features (Figure 1C–E and Figure S2C,D). An independently established line from a second donor (2#) exhibited comparable lineage characteristics (Figure S1A–D). Together, these findings confirm the establishment of TOs with characteristic architecture and trophoblast lineage features.

### 2.2. Bile Acid Mixture Exposure Alters Organoid Architecture and Cytotrophoblast-Associated Features

**Figure 2:**
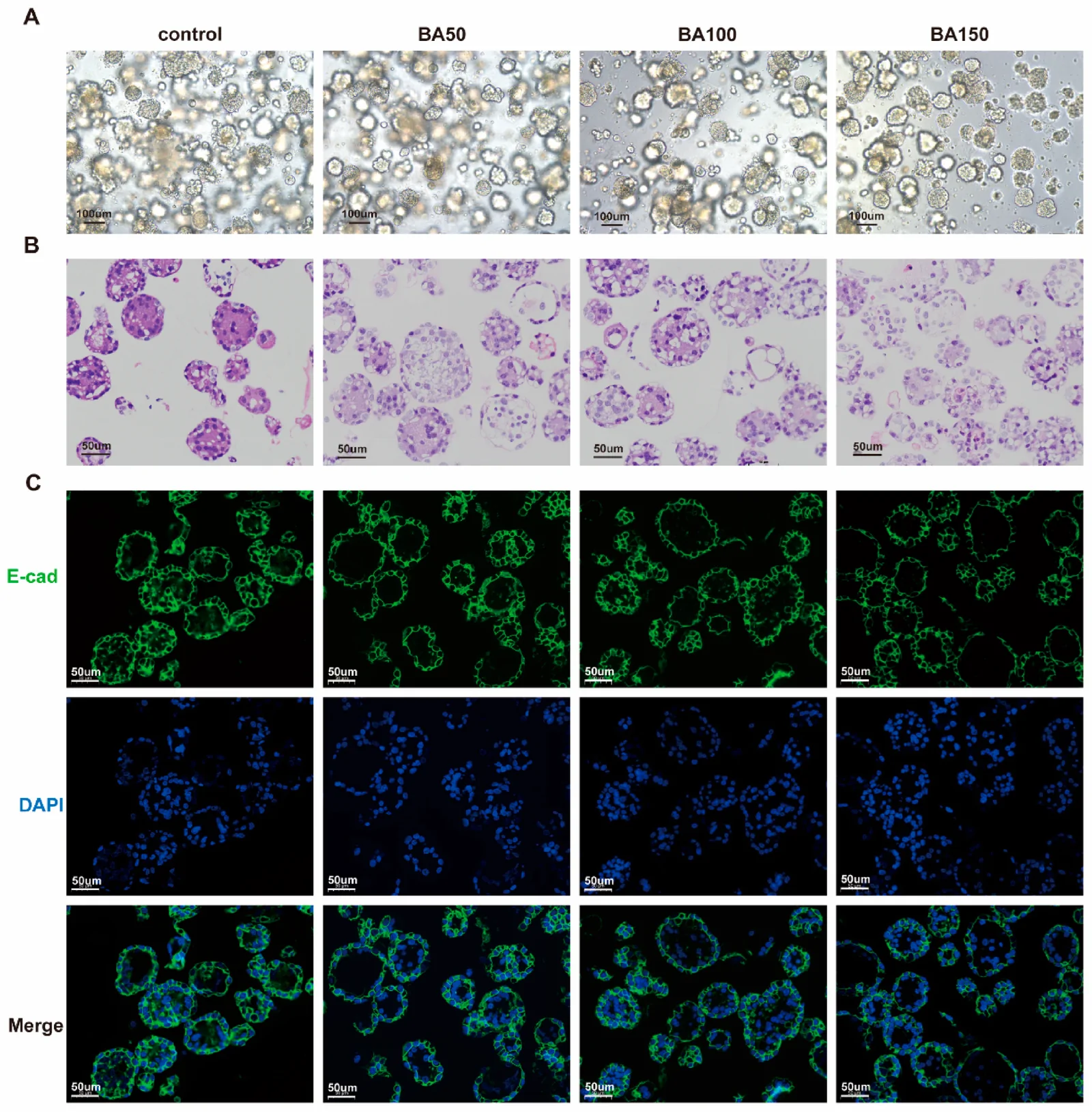
Bile acid (BA) exposure alters trophoblast organoid architecture and cellular organization. TO line 1# was treated with vehicle or a BA mixture at total concentrations of 50, 100, or 150 μM for 96 h. (**A**) Representative bright-field images showing the morphology of vehicle- and BA-treated trophoblast organoids. Scale bars, 100 μm. (**B**) Representative hematoxylin and eosin (H&E)-stained sections showing the histological architecture of trophoblast organoids following BA exposure. Scale bars, 50 μm. (**C**) Representative immunofluorescence images of E-cadherin in vehicle- and BA-treated organoids. E-cadherin is shown in green, and nuclei were counterstained with 4′,6-diamidino-2-phenylindole (DAPI; blue). Scale bars, 50 μm. BA50, BA100, and BA150 denote total BA concentrations of 50, 100, and 150 μM, respectively.

TOs were exposed to the BA cocktail for 96 h. Bright-field imaging showed a concentration-associated reduction in apparent organoid density and increased morphological heterogeneity (Figure 2A). H&E staining showed greater apparent disruption of organoid architecture at higher BA concentrations (Figure 2B). Vehicle-treated organoids were compact and cohesive, whereas BA-treated organoids exhibited vacuole-like or irregular cavity-like spaces and cytoplasmic rarefaction. At 100 and 150 μM BA, epithelial layers appeared thinner, with focal discontinuities, detachment, and loss of integrity. Similar morphological and histological alterations were observed in organoids derived from a second donor (Figure S2A,B).

**Figure 3:**
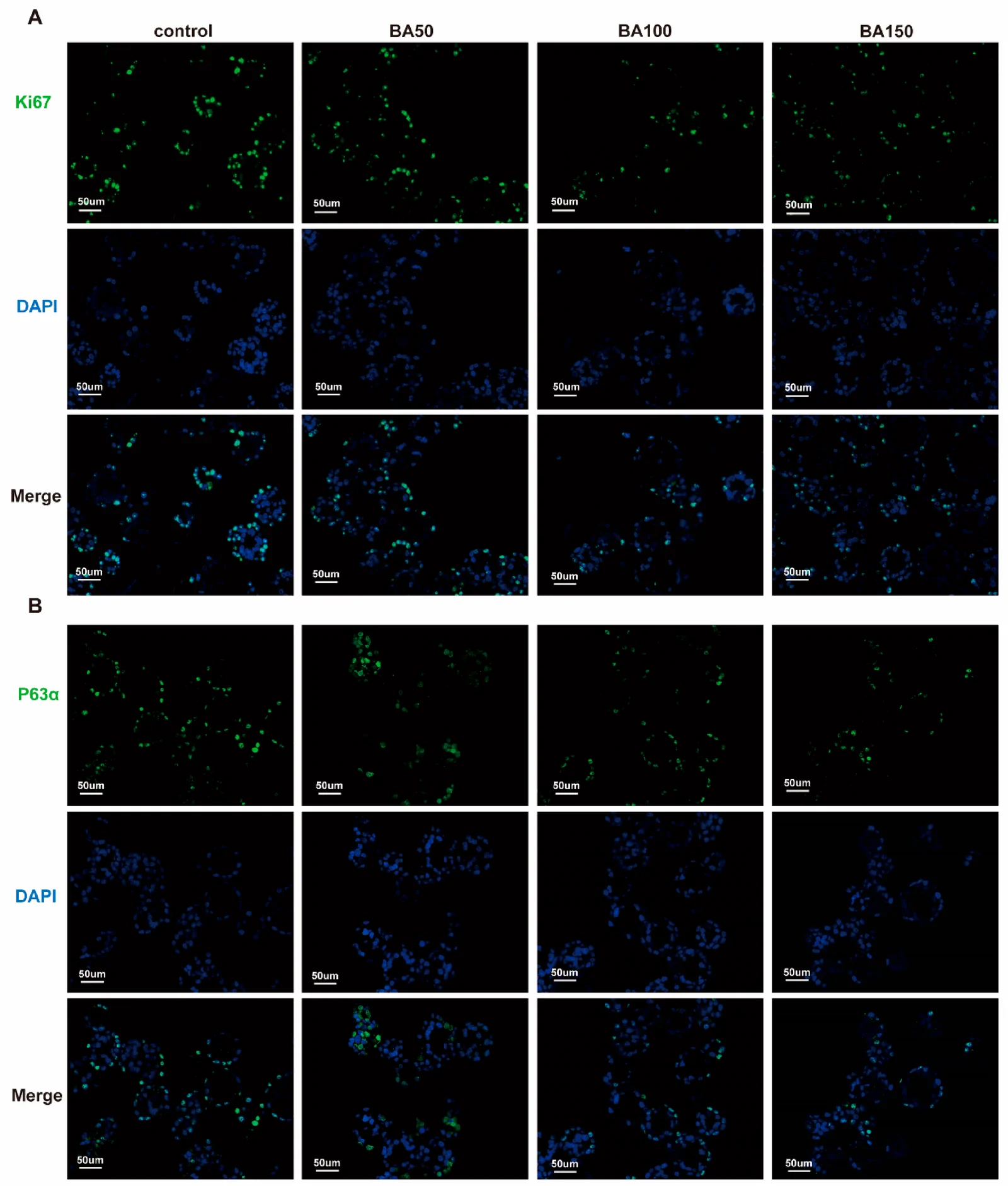
Bile acid (BA) exposure attenuates proliferative and cytotrophoblast progenitor-associated features in trophoblast organoids. TO line 1# was treated with vehicle or a BA mixture at total concentrations of 50, 100, or 150 μM for 96 h. (**A**) Representative immunofluorescence images of Ki-67-positive cells following BA exposure. Ki-67 is shown in green, and nuclei were counterstained with DAPI (blue). Scale bars, 50 μm. (**B**) Representative immunofluorescence images of p63α-positive cells in vehicle- and BA-treated trophoblast organoids. p63α is shown in green, and nuclei were counterstained with 4′,6-diamidino-2-phenylindole (DAPI; blue). Scale bars, 50 μm. BA50, BA100, and BA150 denote total BA concentrations of 50, 100, and 150 μM, respectively.

E-cadherin staining clearly delineated cell–cell boundaries in vehicle-treated organoids (Figure 2C). Following BA exposure, particularly at 100 and 150 μM, these boundaries became discontinuous or were lost, and continuous multinucleated regions were observed. SDC1, another STB-associated marker, also showed more prominent staining in BA150-treated organoids from both donors (Figure S2C,D). Collectively, these qualitative observations indicate altered intercellular organization and STB-associated staining. To assess proliferation-associated features alongside these structural changes, we examined Ki-67, a well-established proliferation marker [15,22]. Ki-67-positive nuclei appeared less abundant in representative sections after BA exposure, particularly at 100 and 150 μM (Figure 3A). p63α-positive nuclei showed a similar qualitative reduction at higher concentrations (Figure 3B), indicating attenuation of proliferative and progenitor-associated features. Similar reductions in Ki-67 and p63α staining were observed in the second donor-derived line at 150 μM BA (Figure S3A,B). Overall, BA mixture exposure disrupted TO architecture and reduced cytotrophoblast-associated proliferative features. Because these imaging observations were qualitative, they do not provide estimates of marker-positive cell fractions or establish increased trophoblast fusion.

### 2.3. Bile Acid Exposure Increases DNA Fragmentation and Reduces ATP-Dependent Viability in TOs

**Figure 4:**
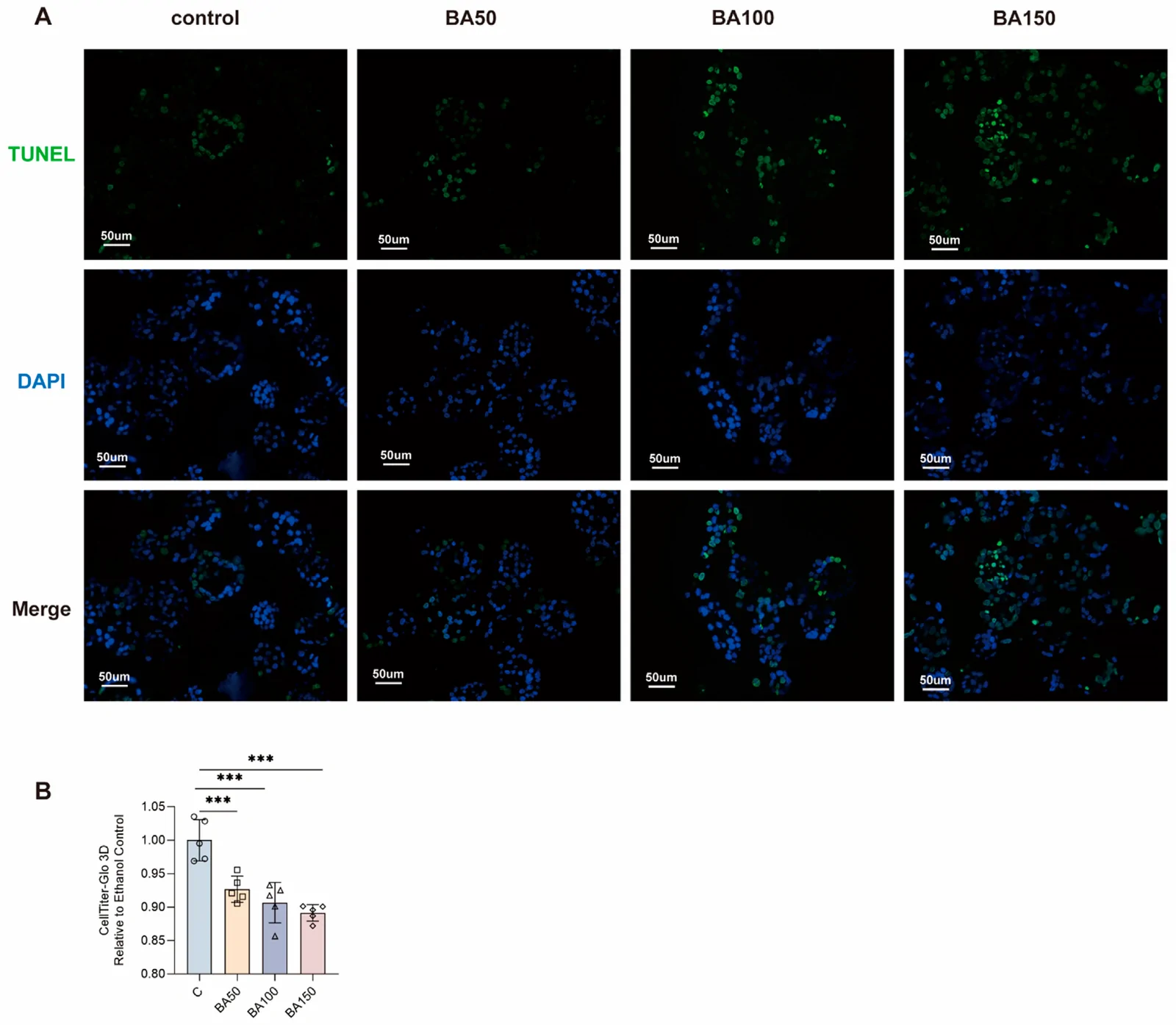
Bile acid mixture exposure increases DNA fragmentation and reduces ATP-dependent viability in trophoblast organoids. Both panels show TO line 1#. (**A**) Representative TUNEL images after 96 h of treatment with vehicle or 50, 100, or 150 μM total BA. TUNEL-positive nuclei are shown in green, and DAPI-counterstained nuclei are shown in blue. Scale bars, 50 μm. (**B**) CellTiter-Glo 3D luminescence after 72 h, normalized to the ethanol vehicle control. Five independent culture replicates per condition were obtained across three experimental runs (*n* = 5). Each data point represents an independent culture replicate. Data are presented as mean ± SD. Statistical comparisons were performed using one-way ANOVA followed by Dunnett’s multiple-comparisons test, with each BA-treated group compared with the vehicle control. *** *p* < 0.001. Dunnett-adjusted *p* values for BA50, BA100, and BA150 versus vehicle were 0.0007, <0.0001, and <0.0001, respectively. BA, bile acid; TO, trophoblast organoid; TUNEL, terminal deoxynucleotidyl transferase dUTP nick-end labeling; SD, standard deviation.

We next examined whether these structural alterations were accompanied by cellular injury. Following 96 h of BA exposure, TUNEL staining became more prominent at higher concentrations (Figure 4A). Cleaved caspase-3 staining was likewise increased in BA150-treated organoids from both donors (Figure S4A,B), supporting an apoptotic component of BA-associated injury. CellTiter-Glo 3D assays further showed reduced ATP-dependent viability after 72 h of BA exposure (Figure 4B); supplementary measurements in line 2# showed similar reductions at both 72 and 96 h (Figure S4C).

### 2.4. Bile Acid Exposure Is Associated with a Predominantly Downregulated Transcriptional Response

**Figure 5:**
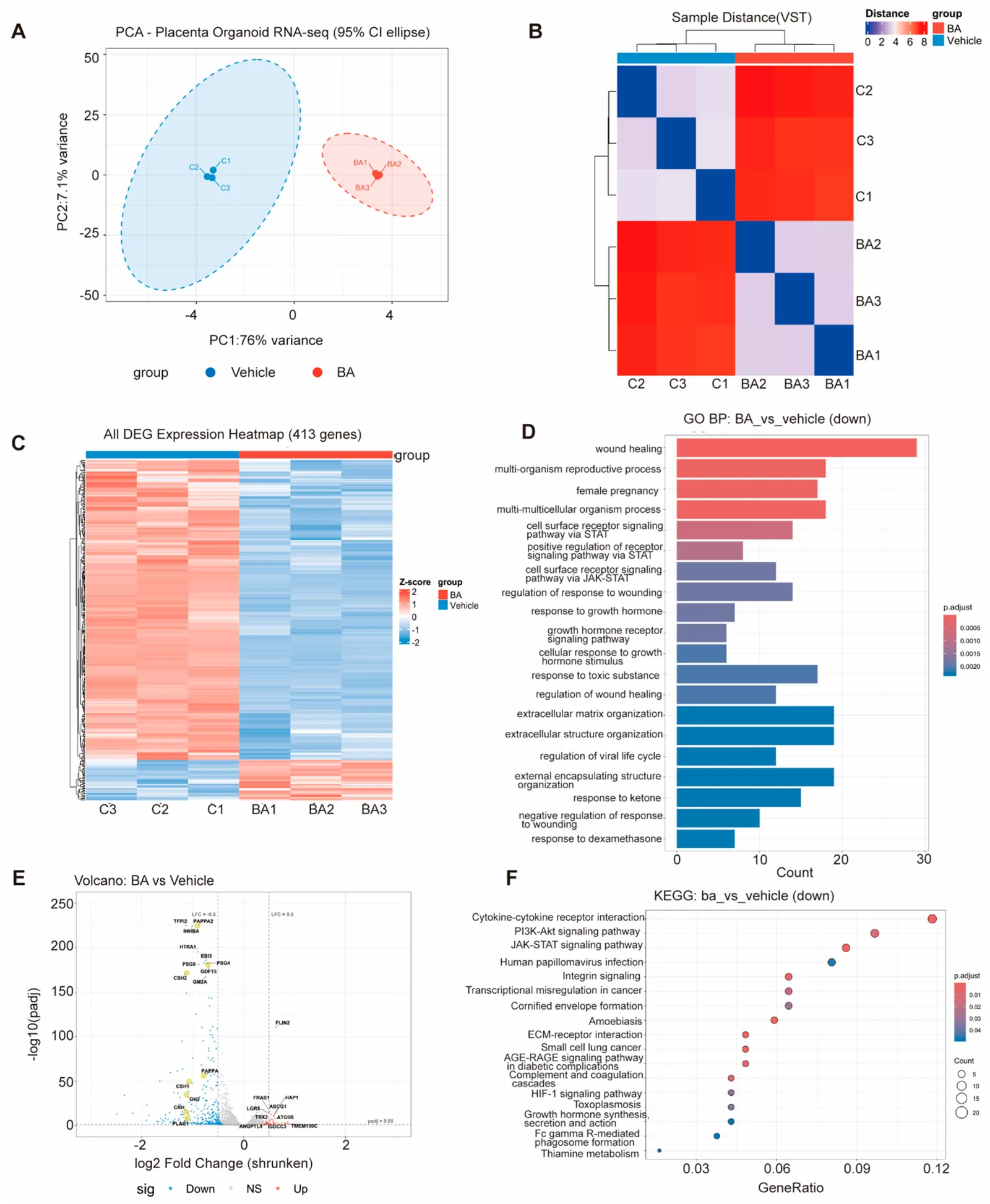
Bile acid exposure is associated with a predominantly downregulated transcriptional response in trophoblast organoids. Bulk RNA-seq was performed in TO line 1# with three independently cultured replicates per condition (one placental donor), using control (C1-C3; *n* = 3 indicated three independent culture replicates in TO line 1#) and BA-treated (BA1-BA3; *n* = 3 indicated three independent culture replicates in TO line 1#) samples following 150 μM BA exposure for 96 h. (**A**) Principal component analysis (PCA). (**B**) Sample-distance heatmap. (**C**) Heatmap of 413 differentially expressed genes (DEGs). (**D**) Gene Ontology (GO) enrichment analysis of downregulated genes. (**E**) Volcano plot of differential gene expression. (**F**) Kyoto Encyclopedia of Genes and Genomes (KEGG) pathway enrichment analysis of downregulated genes.

To characterize the transcriptional response to BA exposure, we performed bulk RNA sequencing of TOs treated with 150 μM BA for 96 h. Principal component analysis (PCA) and sample-distance analysis showed clear separation between BA-treated and vehicle-control samples (Figure 5A,B). Differential expression analysis identified 413 differentially expressed genes (DEGs; Table S4), of which 364 were downregulated and 49 were upregulated (Figure 5C,E). Downregulated transcripts were associated with placental endocrine and secretory functions, including CSH2, as well as epithelial organization, cell adhesion, and transport. Functional enrichment analysis further identified terms related to extracellular matrix organization and growth hormone responses (Figure 5D,F). In contrast, a smaller subset of upregulated genes was associated with lipid handling, including PLIN2. Consistent with the RNA-seq findings, RT-qPCR showed concordant upregulation of the lipid-handling genes PLIN2 and ABCG1 in two independent donor-derived TO lines, although other transcriptional responses differed between donors (Figure S4D).

## 3. Discussion

Although elevated bile acids are implicated in placental dysfunction, their effects on human trophoblasts in a three-dimensional context remain incompletely understood. Here, BA exposure disrupted TO architecture and reduced ATP-dependent viability. These phenotypic changes were accompanied by predominant downregulation of placental function-associated transcripts and selective upregulation of lipid-handling genes. Organoids from an independent donor exhibited similar injury-associated changes and concordant expression changes for selected genes. These findings extend previous observations from two-dimensional trophoblast cultures, placental tissues and explants, and animal models [11,12,13,23,24] into a human three-dimensional context.

A prominent response to BA exposure was the disruption of organoid architecture, characterized by reduced cellular cohesion, cavity-like spaces, and focal loss of epithelial integrity, particularly at higher BA concentrations. These observations are consistent with accumulating evidence that excessive BA exposure compromises trophoblast survival, including reports of reduced viability and increased cell death in human trophoblast models and experimental cholestasis [11,23,25]. Placentas from ICP pregnancies likewise exhibit altered villous architecture, including increased syncytial knots and changes in terminal villous and capillary morphology; notably, several of these abnormalities can be reproduced by exposing human placental explants to cholestatic BAs [26,27]. Although such villous-level changes are not directly equivalent to the epithelial alterations observed in TOs, both point to disruption of placental architecture under cholestatic conditions. In parallel with these structural alterations, BA exposure disrupted E-cadherin-defined cell boundaries, with multinucleated syncytial-like regions and more prominent SDC1 staining. Previous studies have linked E-cadherin remodeling to trophoblast fusion [28,29], while increased SDC1 expression has been associated with syncytial differentiation [30,31]. However, the concurrent downregulation of STB-associated transcripts indicates that these observations cannot be attributed solely to enhanced syncytial differentiation. Overall, these findings extend previous evidence of BA-associated placental injury to a three-dimensional human trophoblast model by demonstrating epithelial remodeling alongside altered STB-associated features. Nevertheless, the qualitative imaging data establish neither an increased fusion rate nor enhanced hormone secretion.

BA exposure also affected features associated with CTB maintenance and survival. Both donor-derived TO lines showed apparently reduced Ki-67 and p63α staining at higher BA concentrations. This concordant attenuation is relevant because p63 is selectively expressed in proliferative villous CTBs and contributes to maintenance of a proliferative, stem cell-like state [32]. The parallel staining changes are consistent with altered proliferation-associated features, given the roles of these markers in the CTB compartment [15,33]. These observations were accompanied by TUNEL-positive DNA fragmentation, cleaved caspase-3 staining, and reduced ATP-dependent luminescence, consistent with previous studies linking excessive, particularly hydrophobic, BA exposure to impaired trophoblast survival through endoplasmic reticulum stress [14,25], mitochondrial dysfunction [13], defective autophagic flux [11], and programmed cell-death pathways [23]. Together, these findings suggest that BA stress disrupts trophoblast homeostasis by attenuating CTB-associated proliferative features and promoting cellular injury, with an apoptotic component. At the transcriptional level, BA exposure was associated with predominant downregulation of genes involved in placental endocrine and secretory processes, epithelial organization, cell adhesion, and transport. These changes are consistent with the accompanying structural alterations and previous evidence of impaired trophoblast adhesion and extracellular matrix signaling under BA stress [34], as well as altered placental BA transporter expression in ICP [35]. In contrast, a smaller group of lipid-handling genes was upregulated, consistent with lipidomic and multi-omics evidence of disturbed placental lipid metabolism in ICP [36,37,38,39]. Notably, PLIN2 and ABCG1 were consistently upregulated in both donor-derived TO lines, although other transcriptional responses varied. Compared with whole-placenta studies highlighting inflammatory and vascular pathways in ICP [40,41], the transcriptional profile observed in TOs may reflect differences in gestational stage or cellular composition [42]. Given the concurrent cellular injury, these bulk transcriptional changes may reflect both altered trophoblast cell states and shifts in cell population composition. Bulk RNA-seq measures transcript abundance rather than pathway activity, hormone secretion, or transport capacity. RT-qPCR confirmed concordant upregulation of PLIN2 and ABCG1 in the two donor-derived lines, whereas the reduction in CSH2 was not reproduced in line 2# (Figure S4D). Thus, these bulk measurements support selected transcriptional responses but cannot resolve cell-type-specific mechanisms.

This study has several limitations. First, first-trimester placenta-derived TOs exposed to a defined BA mixture may not fully recapitulate the gestational environment or heterogeneous BA profiles associated with ICP. Moreover, the apical-in polarity of conventional TOs differs from the maternal-facing orientation of STBs in vivo [43]; BA penetration into the inner organoid compartment was not directly assessed. Although both donor-derived TO lines showed similar injury-associated changes, transcriptomic profiling was restricted to one line. Moreover, bulk RNA-seq cannot distinguish cell-intrinsic transcriptional responses from changes in cell population composition or survival. Furthermore, morphological and immunofluorescence findings were assessed qualitatively. ATP-dependent luminescence was not calibrated against direct cell counts under BA treatment and therefore cannot distinguish a reduction in viable cell number from reduced ATP content per cell. Trophoblast differentiation and placental functional changes also require further validation. Future studies incorporating additional donors, quantitative phenotypic analyses, cell-resolved transcriptomics, and physiologically representative placental models will be needed to clarify the mechanisms underlying BA-associated trophoblast injury.

## 4. Materials and Methods

### 4.1. Placental Tissue Collection

Human first-trimester placental villous tissues (gestational age, 6–10 weeks) were obtained from healthy participants undergoing elective pregnancy termination at International Peace Maternity and Child Health Hospital. Two independent donor-derived TO lines, designated 1# and 2#, were established and used in this study. Gestational age was determined from the last menstrual period. Written informed consent was obtained from all participants. The study was approved by the Ethics Committee of International Peace Maternity and Child Health Hospital (approval number GKLW2023-006). Each tissue sample was collected in ice-cold sterile D-PBS containing 2% penicillin–streptomycin and processed within 2 h.

### 4.2. Establishment and Culture of Human Trophoblast Organoids

Trophoblast organoids (TOs) were established and cultured according to a published protocol, with modifications [17]. Briefly, villous tissue was washed in D-PBS containing 2% penicillin–streptomycin to remove residual blood and minced into approximately 1-mm fragments. Tissue fragments were enzymatically dissociated in a 1:1 (*v*/*v*) mixture of Accutase (A1110501, Thermo Fisher Scientific, Inc., Waltham, MA, USA) and TrypLE Express (12604021, Thermo Fisher Scientific, Inc., Waltham, MA, USA) at 37 °C for 10–15 min, with gentle trituration at 5-min intervals. Digestion was terminated by adding D-PBS containing 10% fetal bovine serum. The resulting cell suspension was filtered through a 100-μm mesh, centrifuged, resuspended in Matrigel (356231, Tewksbury, MA, USA), and plated as 25-μL domes in 24-well plates at an appropriate cell density. TOs were cultured in trophoblast organoid medium (TOM) at 37 °C in a humidified atmosphere containing 5% CO_2_. TOM consisted of Advanced DMEM/F-12 supplemented with N2, B27, penicillin–streptomycin, GlutaMAX, primocin, growth factors (hFGF2, hEGF, R-spondin 1, and hHGF), and small molecules (N-acetyl-L-cysteine, Y-27632, CHIR99021, PGE2, and A83-01). The final concentrations, manufacturers, and catalog numbers of all components are provided in Table S1. The medium was replaced every 2–3 days. For passaging, organoids were recovered from Matrigel using ice-cold D-PBS, dissociated with TrypLE Express and Accutase (2:1, *v*/*v*), and replated in fresh Matrigel. TOs at passages 3–10 were used for all experiments, with passage numbers matched between control and treatment groups within each experiment.

### 4.3. Bile Acid Mixture Treatment

After passage, TOs were maintained under standard culture conditions for 3–4 days before BA exposure. A six-component BA mixture was formulated on the basis of previously reported human plasma BA profiles and experimental mixture formulations [44,45]. The formulation was designed to provide a reproducible mixed-BA stress condition. The cocktail contained CA, CDCA, DCA, LCA, UDCA, and GCDCA at molar proportions of 16.55%, 25.83%, 19.37%, 0.32%, 5.65%, and 32.28%, respectively. Stock component concentrations are listed in Table S2. Total BA concentrations of 50, 100, and 150 μM were selected to examine responses across an increasing exposure range that encompasses clinically elevated maternal serum BA concentrations reported in ICP [6]. Organoids were treated with vehicle or the indicated BA concentrations for 72 or 96 h, depending on the experimental endpoint. The primary ATP-dependent viability assay was performed at 72 h; additional ATP measurements were obtained at both 72 and 96 h. Morphological, histological, immunofluorescence, and TUNEL analyses were performed after 96 h to characterize structural and cellular alterations. RNA-seq and RT-qPCR were also performed after 96 h of treatment with 150 μM BA. Thus, the 72-h assay provided an earlier ATP-related readout, whereas the 96-h analyses assessed subsequent structural, cellular, and transcriptional changes.

### 4.4. Agarose-Assisted Paraffin Embedding of Trophoblast Organoids

Fixed trophoblast organoids were embedded in 3% molten agarose and allowed to solidify. Agarose blocks were trimmed, placed in processing cassettes, dehydrated through graded ethanol, cleared with alcohol–xylene transition solution and xylene, and infiltrated with paraffin at 62 °C. Blocks were embedded in paraffin, cooled at −20 °C, trimmed, and sectioned at 5 μm.

### 4.5. Hematoxylin and Eosin Staining

Paraffin sections were deparaffinized, rehydrated through graded ethanol, and stained using an H&E staining kit (Servicebio, G1076) according to the manufacturer’s instructions. Sections were stained with hematoxylin, differentiated, blued, counterstained with eosin, dehydrated, cleared in xylene, mounted with neutral resin, and examined by bright-field microscopy.

### 4.6. Immunofluorescence Staining

Paraffin sections were deparaffinized, rehydrated, and subjected to microwave-based antigen retrieval in 1× retrieval buffer (boiling at high power followed by 10–15 min at low power). After cooling, sections were washed with PBS, incubated with 3% H_2_O_2_ for 25 min in the dark, and blocked with 3% bovine serum albumin or normal serum for 30 min.

Sections were incubated overnight at 4 °C with primary antibodies against p63α (F10N6, Selleck, Shanghai, China; #F0767; 1:200), Ki-67 (D2H10, Cell Signaling Technology, Danvers, MA, USA; #9027; 1:1000), E-cadherin (24E10, Cell Signaling Technology, #3195; 1:800), cleaved caspase-3 (Cell Signaling Technology, #9661; 1:1500), ITGA6 (Abcam, Boston, MA, USA; ab181551; 1:1000), hCGβ (Abcam, ab324382; 1:200), and SDC1 (Abcam, ab130405; 1:200). After washing, horseradish peroxidase (HRP)-conjugated anti-rabbit or anti-mouse secondary antibodies (Wasci, Shanghai, China; WAS12011) were applied for 50 min at room temperature, followed by tyramide signal amplification (TSA) fluorophore labeling (Wasci, WAS10022; 1:100) for 10 min in the dark. For multiplex staining, antigen retrieval and the subsequent staining procedure were repeated for each additional marker. Sections were counterstained with DAPI (Wasci, WAS13011), mounted with antifade medium (Wasci, WAS14011), and imaged using a fluorescence scanner (3DHISTECH, Budapest, Hungary; Pannoramic MIDI II) under identical acquisition settings.

### 4.7. TUNEL Staining

DNA fragmentation was evaluated using a TUNEL assay kit (Wasci, WAS40011). Paraffin sections were deparaffinized, rehydrated, and incubated with proteinase K working solution at 37 °C for 22 min. A total of 50 μL of TUNEL reaction mixture, prepared by mixing terminal deoxynucleotidyl transferase enzyme and fluorescent labeling solution at a 1:9 ratio, was applied to each section and incubated at 37 °C for 60 min. Sections were washed and counterstained with DAPI (Wasci, WAS13011), mounted with mounting medium (Wasci, WAS14011), and imaged using a fluorescence scanner (3DHISTECH, Pannoramic MIDI II).

### 4.8. Organoid Viability Assay

ATP-dependent luminescence was measured using the CellTiter-Glo^®^ 3D Cell Viability Assay kit (Promega, Madison, WA, USA; G9683) according to the manufacturer’s instructions. Following BA exposure, an equal volume of assay reagent was added to the culture medium. Plates were shaken for 5 min and incubated for an additional 25 min before luminescence was measured using a SpectraMax M3 (Molecular Devices, San Jose, CA, USA). The primary assay was performed in TO line 1# after 72 h of BA exposure, and supplementary measurements were obtained in TO line 2# at 72 and 96 h. Five independent culture replicates per condition were obtained across three experimental runs for each TO line and time point (*n* = 5 per condition). Luminescence values were normalized to the corresponding vehicle controls.

### 4.9. Total RNA Extraction

Total RNA was isolated from trophoblast organoids using the Quick-RNA™ Miniprep Kit (Zymo Research, Orange, CA, USA; R1054) according to the manufacturer’s instructions, including an on-column DNase I digestion step. Organoids were recovered from Matrigel, pelleted, and lysed in RNA Lysis Buffer. Lysates were passed through a Spin-Away™ Filter, mixed 1:1 with 95–100% ethanol, and loaded onto Zymo-Spin™ IIICG columns. After DNase I treatment for 15 min at room temperature, the columns were washed sequentially with RNA Prep Buffer and RNA Wash Buffer. RNA was eluted in 50 μL of DNase/RNase-free water and stored at −80 °C. RNA concentration and purity were determined using a Thermo Scientific NanoDrop 2000c UV–Vis spectrophotometer (Waltham, MA, USA).

### 4.10. Reverse Transcription and Quantitative Real-Time PCR

cDNA was synthesized from 1 μg total RNA using the Hifair^®^ III 1st Strand cDNA Synthesis SuperMix for qPCR with gDNA digester (Yeasen Biotechnology, Shanghai, China; 11141ES60), according to the manufacturer’s instructions. Briefly, RNA was treated with 5× gDNA Digester Mix at 42 °C for 2 min, followed by reverse transcription at 25 °C for 5 min, 55 °C for 15 min, and 85 °C for 5 min. cDNA was used immediately or stored at −80 °C.

Quantitative real-time PCR (qPCR) was performed using the QuantiNova SYBR Green PCR Kit (QIAGEN, Hilden, Germany; 4993626) on a QuantStudio 7 Flex system. Reactions were prepared in a final volume of 10 μL containing 2× SYBR Green Master Mix, ROX reference dye, cDNA, gene-specific primers, and RNase-free water. The cycling conditions were 95 °C for 2 min, followed by 40 cycles of 95 °C for 10 s and 60 °C for 30 s. RNA from TO lines 1# and 2# was analyzed separately after vehicle or 150 μM BA treatment for 96 h. Three independent culture replicates were analyzed per condition for each TO line (*n* = 3 per condition). The target genes included PAGE4, TEAD4, MSX2, GATA2, CDH1, TJP1, CSH2, PLIN2, ABCG1, and ANGPTL4. Target gene expression was normalized to three reference genes (ACTB, YWHAZ, and TOP1), using the arithmetic mean of their Ct values for each sample. Relative expression was calculated using the 2^−ΔΔCt^ method [46], with ΔΔCt determined relative to the mean ΔCt of three corresponding vehicle-control samples within each TO line. Primer sequences are listed in Table S3.

### 4.11. RNA Sequencing and Bioinformatics Analysis

Total RNA was extracted from TOs following vehicle or 150 μM BA treatment for 96 h, with three independently cultured replicates per condition from one donor-derived line (1#), representing within-line culture replication rather than independent donor replication. RNA integrity was assessed using an Agilent 2100 Bioanalyzer (Santa Clara, CA, USA), and all samples had RNA integrity number (RIN) values ≥ 8.5. Sequencing libraries were constructed from 1 μg of total RNA using the NEBNext Ultra II RNA Library Prep Kit (Waltham, MA, USA) with poly(A) enrichment of mRNA. Sequencing was performed by Anoroad Gene Technology (Beijing, China) on the DNBSEQ-T7 platform to generate 150-bp paired-end reads.

Raw reads were subjected to quality control using FastQC (v0.11.9), and adapter sequences and low-quality bases were removed using fastp (v0.23.4). Clean reads were aligned to the human reference genome (GRCh38.109) using HISAT2 version 2.2.1, and gene-level read counts were obtained with HTSeq version 2.0.2. Gene expression was quantified as counts per million mapped reads (CPM). Differential expression analysis between BA-treated and vehicle-control groups was performed using DESeq2 in R. Genes with a Benjamini–Hochberg-adjusted *p* value < 0.05 and |log2 fold change| > 0.5 were defined as differentially expressed genes (DEGs). Gene Ontology (GO) and KEGG pathway enrichment analyses of DEGs were performed using clusterProfiler, with adjusted *p* < 0.05 considered statistically significant.

### 4.12. Statistical Analysis

Statistical analyses were performed using GraphPad Prism 9 and R version 4.4.2. Unless otherwise specified, comparisons between two groups were assessed using two-sided Welch’s *t*-tests, which do not assume equal variances. Comparisons among three or more groups were analyzed using one-way ANOVA, followed by Dunnett’s multiple-comparisons test to compare each BA-treated group with the vehicle control. Adjusted *p* values from Dunnett’s test are reported.

## 5. Conclusions

In conclusion, exposure to a defined BA mixture disrupted TO architecture and attenuated proliferation-associated features, accompanied by increased DNA fragmentation, cleaved caspase-3 staining, and reduced ATP-dependent viability. Transcriptomic profiling revealed predominant downregulation of placental function-associated genes and selective upregulation of lipid-handling genes. Both donor-derived TO lines showed similar phenotypic alterations and concordant expression changes for selected genes. Together, these findings advance our understanding of BA-associated trophoblast injury and its accompanying transcriptional alterations in a human three-dimensional model.

## Data Availability

The raw RNA-sequencing data and processed expression data generated in this study have been deposited in the NCBI Gene Expression Omnibus (GEO) under accession number GSE343936 and will be publicly available upon publication.

## Supplementary Materials

The following supporting information can be downloaded at: https://www.mdpi.com/article/10.3390/ijms272110023/s1.
