# Supplementary material for: Bile Acid Stress Disrupts Homeostasis and Placental Functional Programs in Human First-Trimester Trophoblast Organoids
Source: Int J Mol Sci. 2026 Nov 9;27(21):10023. doi: 10.3390/ijms272110023 (PMC13763841; doi:10.3390/ijms272110023)
Supplement: Supplementary file 1 [file ijms-27-10023-s001.zip › Supplementary Materials-Fig S1-4, Table S1-3.pdf]

Supplementary materials

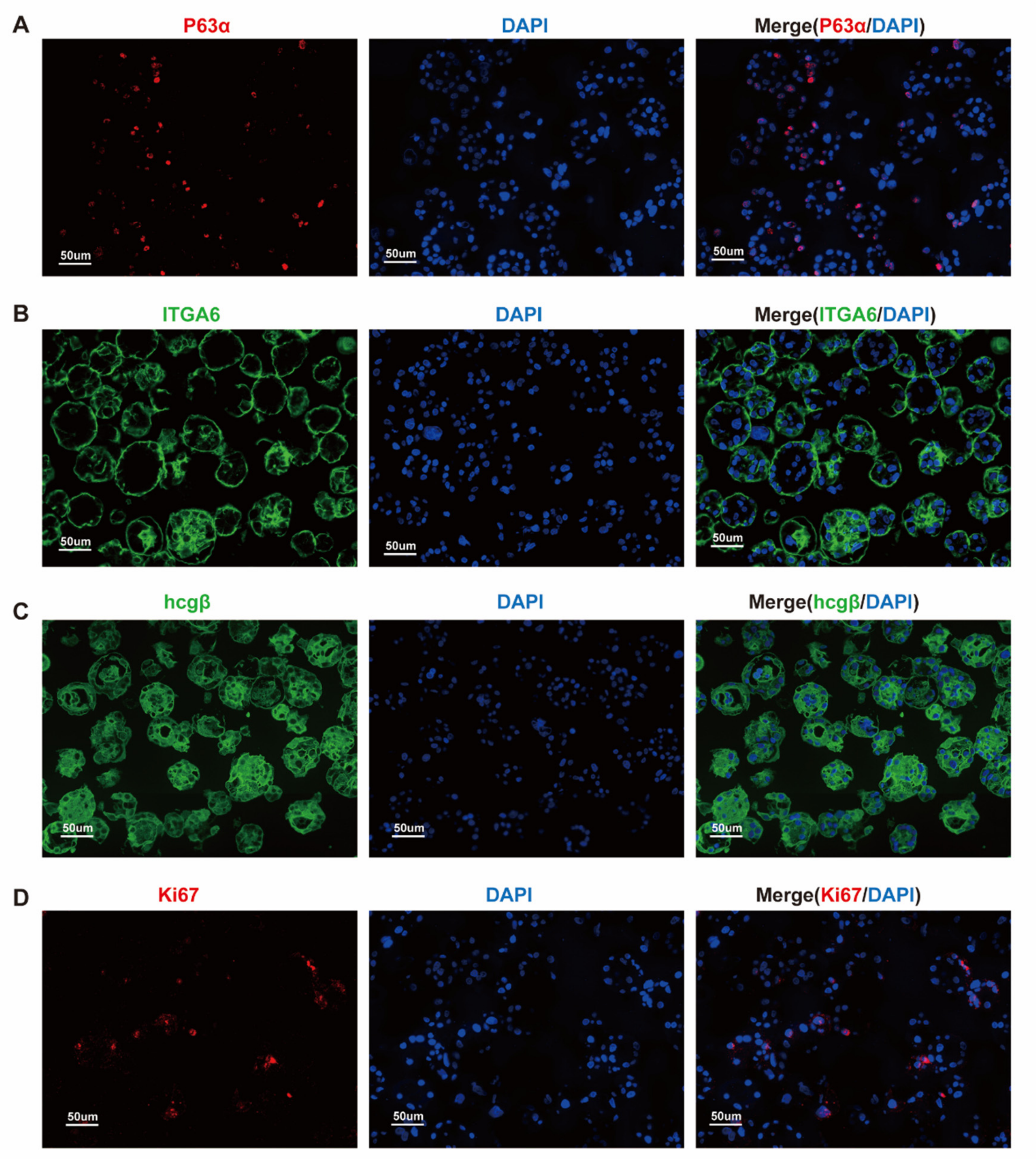

Figure S1. Trophoblast lineage characterization of TO line 2#.

Representative immunofluorescence images of (A) p63α, (B) ITGA6, (C) hCGβ, and (D) Ki-67 in TO line 2#, acquired using a 63× objective. p63α and Ki-67 are shown in red, ITGA6 and hCGβ in green, and DAPI-counterstained nuclei in blue. Scale bars, 50 μm.

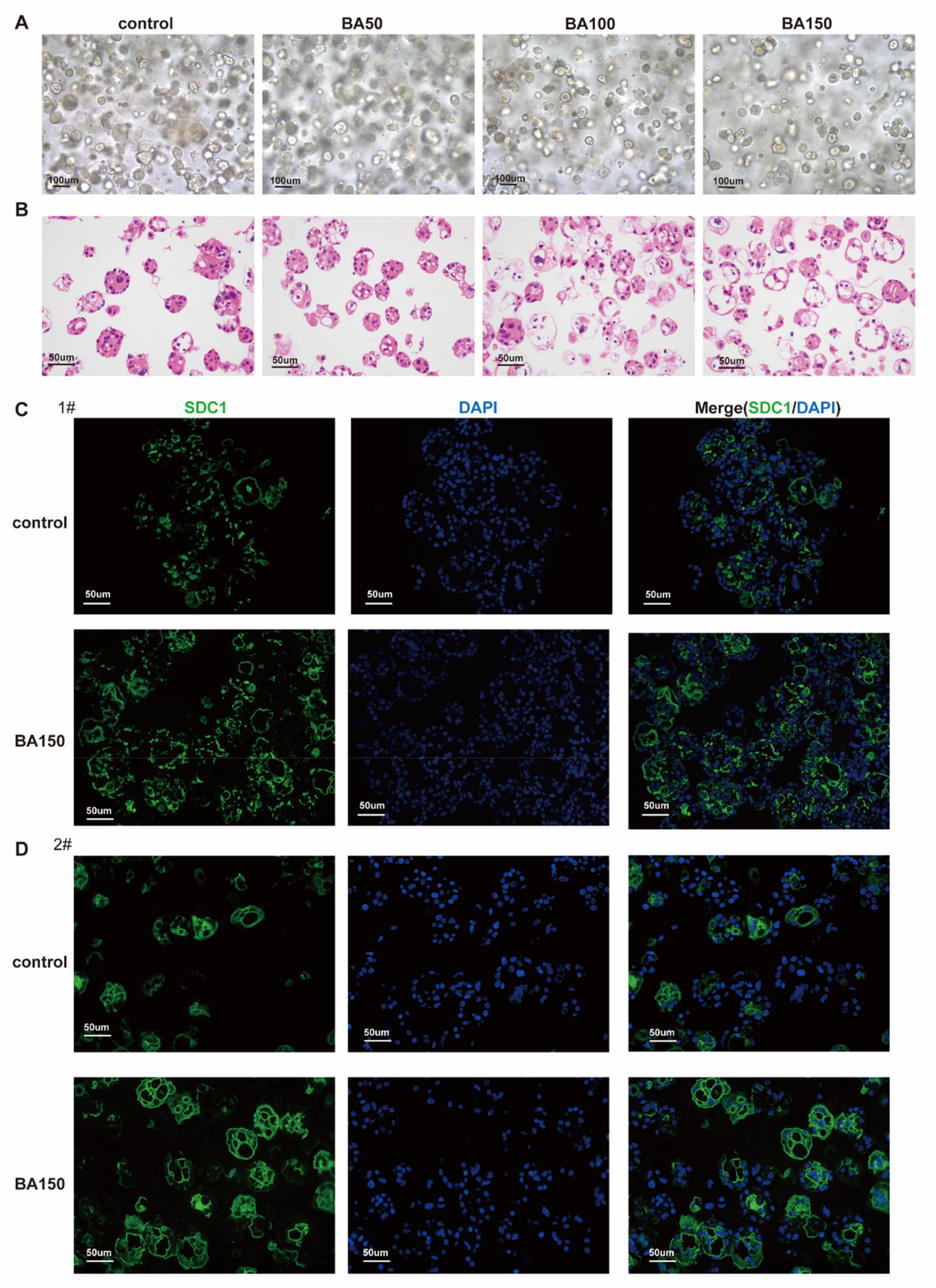

Figure S2. Organoid morphology and SDC1 staining following bile acid exposure.

(A) Representative bright-field images (10× objective) and (B) hematoxylin and eosin (H&E)-stained sections (40× objective) of TO line 2# following vehicle or 50, 100, or 150 μM BA treatment for 96 h. Scale bars, 100 μm (A) and 50 μm (B). (C,D) Representative SDC1 immunofluorescence images in TO lines 1# (C) and 2# (D), respectively, following

vehicle or 150 μM BA treatment for 96 h, acquired using a 63× objective. SDC1 is shown in green and DAPI-counterstained nuclei in blue. Scale bars, 50 μm.

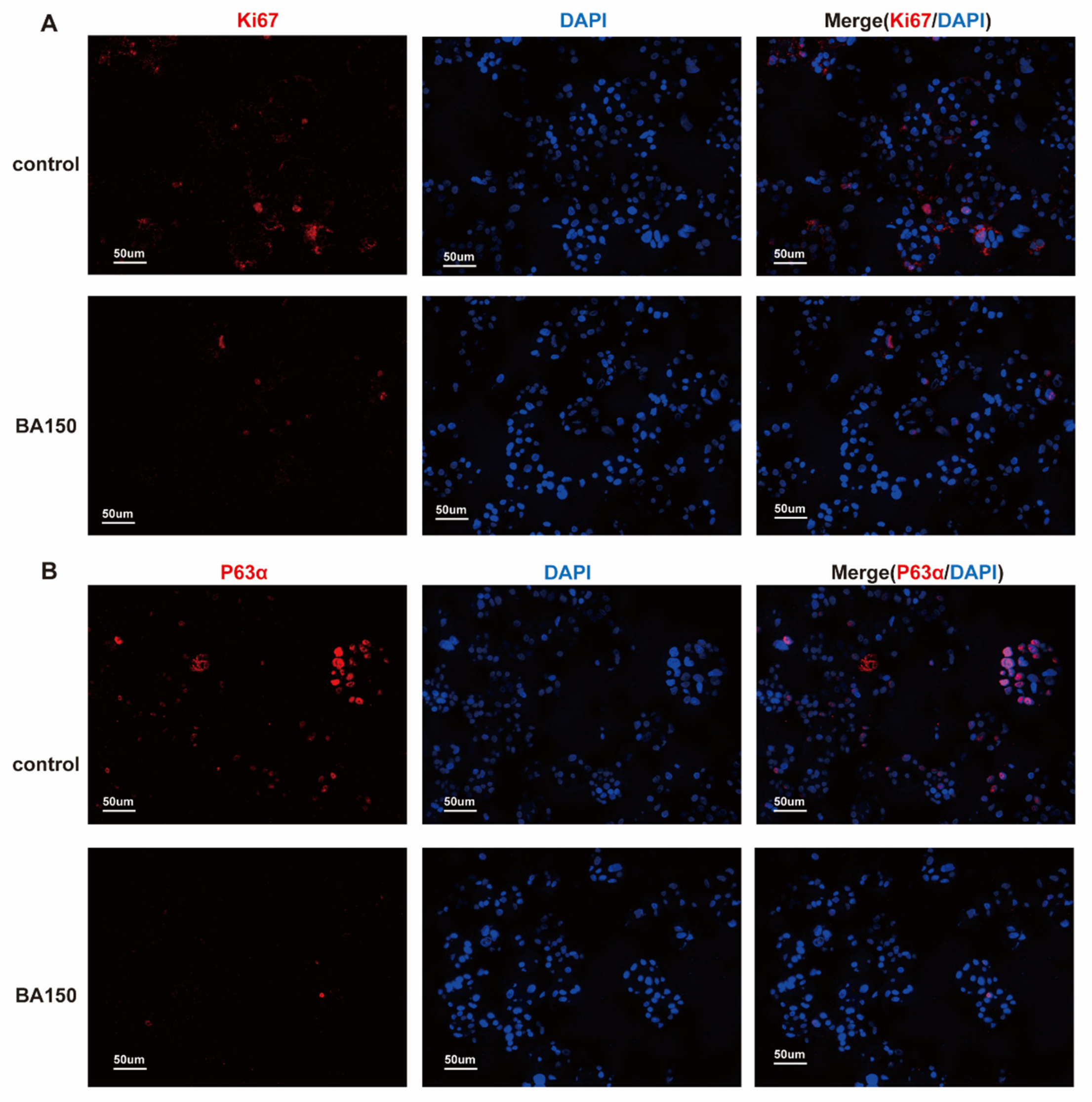

Figure S3. Ki-67 and p63α staining in TO line 2# following bile acid exposure. Representative immunofluorescence images of (A) Ki-67 and (B) p63α in TO line 2# following vehicle or 150 μM BA treatment for 96 h, acquired using a 63× objective. Both markers are shown in red and DAPI-counterstained nuclei in blue. Scale bars, 50 μm.

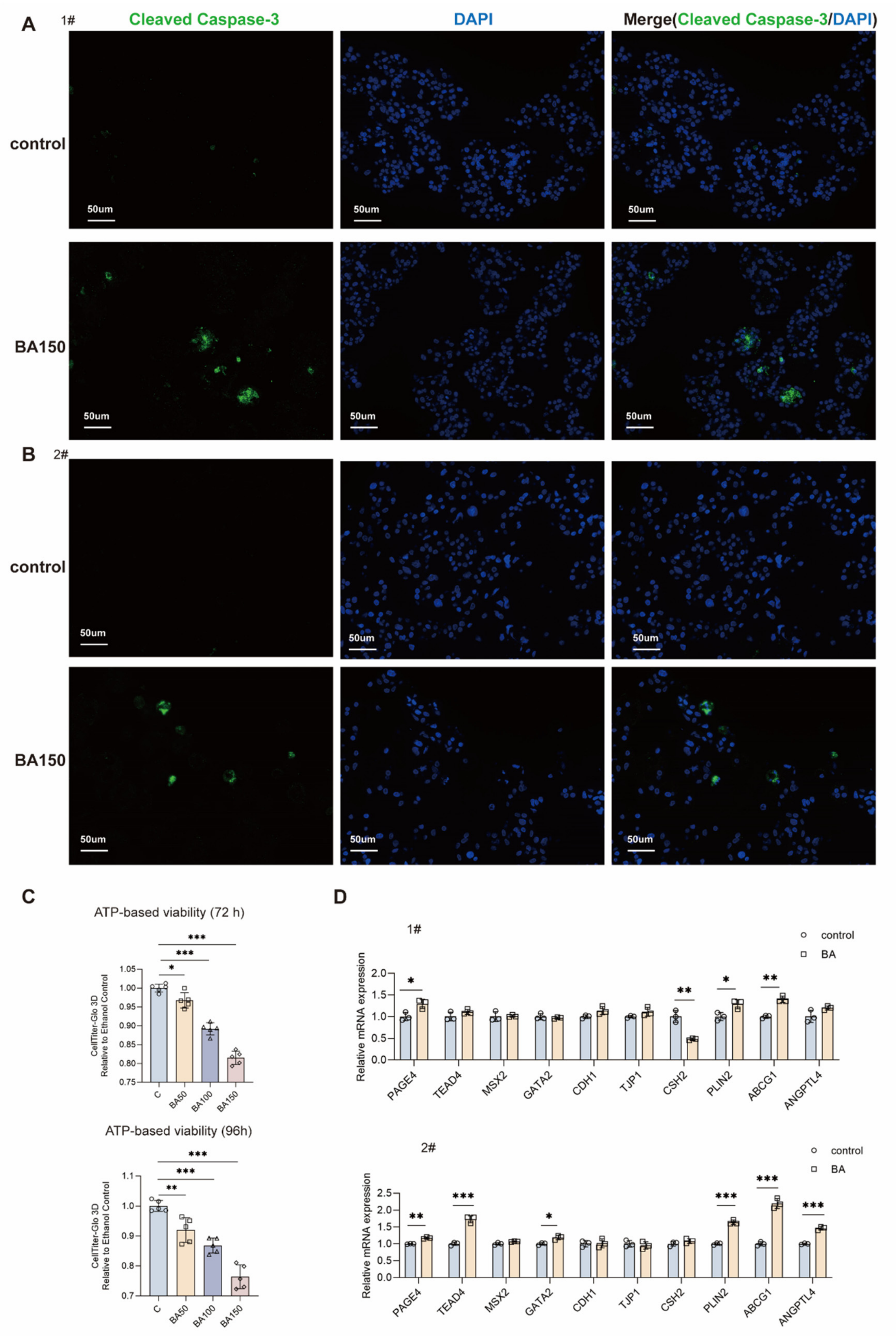

Figure S4. Cleaved caspase-3 staining, ATP-dependent viability, and RT-qPCR analysis following bile acid exposure.

(A,B) Representative immunofluorescence images of cleaved caspase-3 in TO lines 1# (A)

and 2# (B), respectively, following vehicle or 150 μM BA treatment for 96 h, acquired using a 63× objective. Cleaved caspase-3 is shown in green and DAPI-counterstained nuclei in blue. Scale bars, 50 μm. (C) CellTiter-Glo 3D luminescence in TO line 2# after 72 h (upper) and 96 h (lower) of BA exposure, normalized to the corresponding vehicle controls. Five independent culture replicates per condition were obtained across three experimental runs at each time point (n = 5). Each data point represents an independent culture replicate. Data are presented as mean ± SD. Statistical comparisons were performed separately at each time point using one-way repeated-measures ANOVA followed by Dunnett's multiple-comparisons test, with each BA-treated group compared with the vehicle control. Dunnett-adjusted P values for BA50, BA100, and BA150 versus vehicle were 0.0181, <0.0001, and <0.0001 at 72 h, and 0.0035, <0.0001, and <0.0001 at 96 h, respectively. (D) Relative mRNA expression of selected genes in TO lines 1# (upper) and 2# (lower) following vehicle or 150 μM BA treatment for 96 h. Three independent culture replicates were analyzed per condition for each TO line (n = 3). Each data point represents an independent culture replicate. Gene expression was normalized to ACTB, YWHAZ, and TOP1 using the $2^{-\Delta\Delta Ct}$ method. Statistical comparisons were performed using two-sided unpaired Welch's t-tests within each TO line. P values were not adjusted for multiple comparisons. In TO line 1#, significant differences were observed for PAGE4 (P = 0.02452), CSH2 (P = 0.006122), PLIN2 (P = 0.02521), and ABCG1 (P = 0.001219). In TO line 2#, significant differences were observed for PAGE4 (P = 0.003205), TEAD4 (P = 0.0003193), GATA2 (P = 0.01655), PLIN2 (P = 0.0001011), ABCG1 (P = 0.0001254), and ANGPTL4 (P = 0.0006148). Data are presented as mean ± SD. *P < 0.05, **P < 0.01, ***P < 0.001.

Table S1. Composition of trophoblast organoid medium (TOM) used for the establishment and long-term culture of human trophoblast organoids.

Product	Manufacturer	Catalog number	Final concentration
Advanced DMEM/F-12	Meilunbio	PWL038-1	1×
B27 Supplement Minus Vitamin A	Meilunbio	PWL043-1	1×
N2 Supplement	Meilunbio	PWL079-1	1×
Penicillin-Streptomycin	Meilunbio	MA0110-1	1×
GlutaMAX I	Thermo Fisher Scientific	25030081	1×
Primocin	InvivoGen	ant-pm-05	100 μg/mL
Recombinant human FGF2	Novoprotein	C046	100 ng/mL
Recombinant human EGF	PeproTech	AF-100-15-100UG	50 ng/mL
Recombinant human R-spondin 1	Novoprotein	CX83	80 ng/mL
Recombinant human HGF	Novoprotein	CJ72	50 ng/mL
N-acetyl-L-cysteine	Sigma-Aldrich	A9165-25G	1.25 mM
Y-27632	Selleck Chemicals	S1049-10mg	2 μM
CHIR99021	Selleck Chemicals	S2924-5mg	1.5 μM
Prostaglandin E2 (PGE2)	Selleck Chemicals	S3003	2.5 μM
A83-01	Selleck Chemicals	S7692	0.5 μM

Table S2. Composition and preparation details of the bile acid mixture.

No.	Abbrev.	Full name	Manufacturer	Catalog No.	Stock concentration (mM)
1	CA	Cholic acid	Sigma	C1129	16.55
2	CDCA	Chenodeoxycholic acid	Sigma	C9377	25.83
3	DCA	Deoxycholic acid	Sigma	D2510	19.37
4	LCA	Lithocholic acid	Sigma	L6250	0.32
5	UDCA	Ursodeoxycholic acid	Sigma	U5127	5.65
6	GCDCA	Glycochenodeoxycholic acid	Sigma	G0759	32.28
				**Total**	**100**

Table S3. Primers used for RT-qPCR amplification.

Gene	Forward primer (5'-3')	Reverse primer (5'-3')
CSH2	CAAACGCTGATGGGGAGGC	GGTCGGTCACAGGATGCC
PLIN2	ATGGCATCCGTTGCAGTTGAT	GGACATGAGGTCATACGTGGAG
ABCG1	ATTCAGGGACCTTTCCTATTCGG	CTCACCACTATTGAACTTCCCG
ANGPTL4	TGGTTTGGCACCTGCAGCCATTC	TGCTGCCATGGGCTGGATCAAC
PAGE4	CGGTGAATCTCAGCAAGAGGAAC	GCTCACTCCGAGTCTTTTCCAG
TEAD4	GAACGGGGACCCTCCAATG	GCGAGCATACTCTGTCTCAAC
MSX2	GCAGGAACCCGGCCGATATT	CTGACGGAACTTGCGCTCCA
GATA2	AAGGCTCGTTCCTGTTCAGA	GGCATTGCACAGGTAGTGG
CDH1	AAAGGCCCATTTCCTAAAAACCT	TGCGTTCTCTATCCAGAGGCT
TJP1	CAACATACAGTGACGCTTCACA	CACTATTGACGTTTCCCCACTC
ACTB	AGCATCCCCCAAAGTTCACAA	TGGGGTGGCTTTTAGGATGG
YWHAZ	TGATCCCCAATGCTTCACAAG	GCCAAGTAACGGTAGTAATCTCC
TOP1	TCGAAGCGGATTTCCGATTGA	CTTTGTGCCGGTGTTCTCGAT

ACTB, YWHAZ, and TOP1 were used as reference genes.
